# Microfiber-Based Bragg Gratings for Sensing Applications: A Review

**DOI:** 10.3390/s120708861

**Published:** 2012-06-27

**Authors:** Jun-Long Kou, Ming Ding, Jing Feng, Yan-Qing Lu, Fei Xu, Gilberto Brambilla

**Affiliations:** 1 College of Engineering and Applied Sciences and National Laboratory of Solid State Microstructures, Nanjing University, Nanjing 210093, China; E-Mails: koujunlong2006@163.com (J.K.); xf1997@163.com (J.F.); yqlu@nju.edu.cn (Y.L.); 2 Optoelectronics Research Centre, University of Southampton, Southampton, SO17 1BJ, UK; E-Mails: md20g09@orc.soton.ac.uk (M.D.); gb2@orc.soton.ac.uk (G.B.)

**Keywords:** microfiber, grating, sensor, optical fiber component, refractive index, temperature, strain, force, optical fabrication, focused ion beam

## Abstract

Microfiber-based Bragg gratings (MFBGs) are an emerging concept in ultra-small optical fiber sensors. They have attracted great attention among researchers in the fiber sensing area because of their large evanescent field and compactness. In this review, the basic techniques for the fabrication of MFBGs are introduced first. Then, the sensing properties and applications of MFBGs are discussed, including measurement of refractive index (RI), temperature, and strain/force. Finally a summary of selected MFBG sensing elements from previous literature are tabulated.

## 1. Introduction

Fiber Bragg gratings (FBGs) are periodic modulations of the refractive index (RI) along the fiber length. Since their discovery in 1978 [1], FBGs have been widely used as a sensing element in areas including temperature monitoring, strain sensing, rotation sensing, and underwater acoustic sensing. In fact, fiber-based techniques provide a technology which can produce sensors that are immune to electromagnetic interference, inherent self-referencing, lightweight, easily multiplexable, and in most cases have the potential to be produced at low cost. Over the last two decades, FBGs have been manufactured mainly by modifying the core refractive index using interferometric or point-by-point techniques. Most of the interferometric techniques use a phase mask and an ultraviolet (UV) laser [2], typically an excimer laser or a frequency doubled Ar^+^ ion laser. Moreover, FBGs are typically several millimeters long and a hundred micrometers thick. The large size limits its performance and its use in some special applications such as RI sensing and detection of ultra-small objects.

Recently, microfibers (MFs) have attracted great attention because of their low loss, large evanescent fields, strong confinement, configurability, and robustness [3,4]. MFs have found potential applications in a wide range of fields from telecommunications to sensors, and lasers [5–21]. Because of their large evanescent field and micrometer-scale size, MFs are seen as useful tools to exploit FBGs as refractive index sensors and to reduce their size. Initially, microfiber-based Bragg gratings (MFBGs) have been fabricated by etching away the fiber cladding after writing the grating in the photosensitive core [22–25] or by UV irradiation on the MF drawn from a fiber preform [26–30]. Yet, to shorten the grating length and reduce its size, strong RI modulations (∼10^−3^−10^−1^) are necessary. Strong RI modulations can be obtained alternating layers of materials with high contrast RI, such as glass and air. For a normal optical fiber, this process requires the removal of large amounts of material (the propagating mode is confined at a depth > 50 μm from the fiber surface). On the contrary, for a MF, it only requires the removal of small amounts of fused silica because the modal field is comparable to or larger than the MF cross section. MFBGs with strong RI modulation can be fabricated using focused ion beam (FIB) milling [31–38] and can be as short as ∼10^1^–10^2^ μm.

This review will mainly focus on MFBGs written in MFs with diameters smaller than 10 μm. This paper is organized as follows. Fabrication of MFBGs is shown in Section 2. Section 3 is dedicated to the discussion of the MFBGs fundamental characteristics and sensing properties. Section 4 is devoted to the sensing applications previously reported in the literature. Finally, conclusions and future prospects of MFBGs are given in Section 5.

## 2. Fabrication of MFBGs

Several techniques have been reported in the literature for the fabrication of FBGs on MFs and they can be classified as follows:

1. Etch-eroded commercial FBG or UV irradiated FBG [22–30].
2. FIB-milled FBG on MFs [31–38].
3. Femtosecond-laser-irradiated FBG on MFs [39,40].
4. Other techniques [41–44].

**Figure 1.**
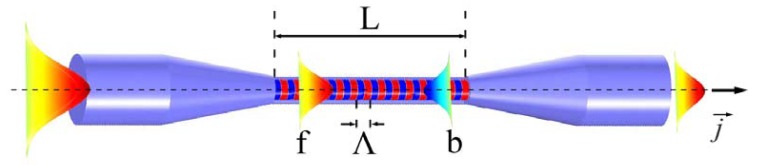
Schematic illustration of an uMFBG. The alternated red and blue stripes represent the refractive index modulation induced by photosensitization treatment. L and Λ are the grating length and period, respectively. f/b represent forward/backward propagating modes.

Both cladding-etched commercial FBGs and UV irradiated FBGs in MFs are uniform MFBGs (uMFBGs), meaning that the grating region experiences only RI modulation and not structural perturbations as indicated in Figure 1.

The most commonly used technique to get an uMFBG is to etch a single mode fiber (SMF) after the FBG has been written in the photosensitive Ge-doped core [22–25]. Usually, a hydrofluoric acid aqueous solution (∼20%–50%) at room temperature is employed for the etching process at an etching speed of ∼0.5–2 μm/min. The diameter of the etched fiber could be measured and controlled *in situ* by monitoring the transmission loss.

### 2.1. Etch-Eroded Commercial FBGs or UV Irradiated FBGs

An alternative way is to use a 248 nm KrF excimer laser and a uniform phase mask to inscribe FBG in MFs drawn from 125 μm-diameter fibers [26,27]. The preform fibers are usually highly Ge-doped and have large cores to guarantee that the MFs have a large enough photosensitive cross section. Sometimes, hydrogen loading treatment are further employed to increase the photosensitivity [27]. However, during the hydrogen loading treatment, high pressures and temperatures are needed, which complicates the procedure [27].

To avoid additional hydrogen loading or photosensitization treatments, Ran *et al.* used a 193 nm ArF excimer laser and phase mask to inscribe strong FBG in MFs drawn from both standard telecom SMF [28] and 62.5/125 μm multimode fiber (MMF) [29]. This method utilizes the high efficiency associated with two-photon excitation at 193 nm [30].

### 2.2. FIB-Milled MFBGs

FIB milling, a powerful micromachining technique, has also been used to fabricate MFBGs [31–38]. This method employs accelerated ions to mill nanometer-scale features on MF surfaces to form corrugated structures. As the index modulation results from changes in the structure, this kind of gratings are called structural MFBGs (sMFBGs).

Prior to the milling, the MF is coated with a thin film of metal, e.g., aluminum or gold [31–35], to prevent charge accumulation which cause ion deflections and large fabrication errors. Alternatively, MF can also be laid on a doped silicon wafer [36]: due to van der Waals' forces, the MF tightly attaches to the conductive substrate and it avoids charging by transferring charges to the wafer.

During the FIB micromachining process, the MF sample should be fixed firmly in the vacuum chamber to minimize sample displacements. A 30 kV, 10–300 pA Ga^+^ ion beam is usually used to get enough milling accuracy depending on different FIB systems. The total milling process takes minutes to hours according to the beam current used and milling area. After the machining process, for a nonmetallic sMFBG, the MF is immersed in metal etchant to totally remove the metal film and then is cleaned with deionized water.

**Figure 2.**
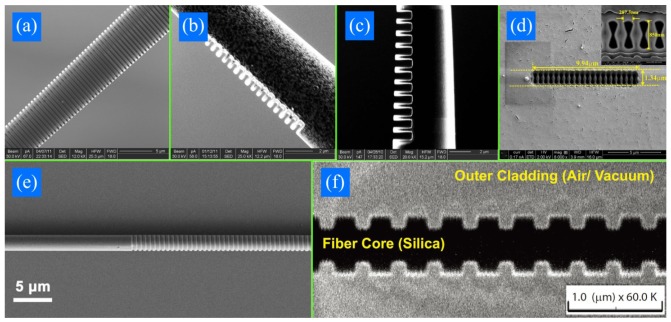
FIB/SEM pictures of gratings fabricated on MF tips (**a**) [31], (**b**) [32], (**c**) [35] and MF tapers (**d**) [34], (**e**) [36], (**f**) [37]. Reprinted with permission. Copyright 2011 Optical Society of America and Copyright 2011 IEEE.

Figure 2 shows FIB/SEM pictures of sMFBGs fabricated from different groups. Gratings in Figure 2(a–c) are fabricated on MF tips while the rest are on tapers. The sMFBGs are fabricated on MFs with diameter ranging from 560 nm (Figure 2(f)) to 6.6 μm (Figure 2(a)) and the number of period of the gratings varies from 11 (Figure 2(c)) to 900 (Figure 2(e)). Both high (5 × 10^−3^−10^−1^, Figure 2(c,d,f)) and low (10^−4^−5 × 10^−3^, Figure 2(a,b,e)) average RI modulations have been achieved by FIB-milled sMFBG. In all, FIB provides researchers a flexible way to get all kinds of structures with high accuracy at will and without additional masks. Yet, batch production cannot be envisaged for this method.

### 2.3. Femtosecond-Laser-Irradiated MFBGs

Femtosecond-laser-irradiation is another way to cause periodically physical deformation on the surface of MFs [40]. During the femtosecond laser irradiation, the ultra-short laser pulse transfers energy to the electrons in the material irradiated through nonlinear ionization [45]. When a sufficiently high energy is achieved, pressure or shock wave will cause melting or non-thermal ionic motion, resulting in permanent structural damages in the material. Aided with proper phase masks, MFBGs can be fabricated on the surface of the MFs [39,40].

### 2.4. Other Techniques

**Figure 3.**
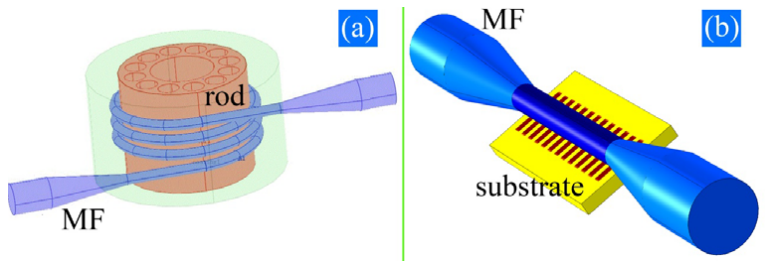
Proposed MFBGs by wrapping a MF on a microstructured rod with an internal channel [41,44] or by laying the MF on a substrate with pre-treated microstructures [47].

In addition to the previous techniques, other methods have also been demonstrated or proposed. MFBGs can be manufactured by wrapping a MF on a microstructured rod with an internal channel (see Figure 3(a)) or by laying the MF on a substrate with pre-treated microstructures (see Figure 3(b)). By exploiting the fraction of power propagating in the periodically distributed patterns in the rod or the substrate, light transmission could be modulated. This compact scheme of Figure 3(a) can be used as a RI sensor when the evanescent field extends in the inner fluidic channel. Both methods avoid post-processing the thin MFs and have great flexibility. However, the MFs have to be coated with low index polymer [46] which means that they are not suitable for high temperature sensing.

Ding *et al.* [42] combined metal lift-off technology with lithography to produce metallic surface gratings, which provided a high and constant sensitivity to the ambient medium RI, while Phan Huy *et al.* [43] demonstrated an improvement in the sensitivity of RI by making use of the suspended core of a microstructured fiber.

## 3. Fundamental Characteristics and Sensing Properties of MFBGs

### 3.1. Fundamental Characteristics of MFBGs

In the grating, the forward (f) and backward (b) propagating modes are related by

(1) $$\vec{\beta_{b}}=\vec{\beta_{f}}+m\frac{2\pi }{\Lambda }\vec{j}.$$

where β_i_ = (2π/λ)n_eff,i_ (i = f or b) is the mode propagation constant, m is the diffraction order, Λ is the period of the grating and *J⃗* is the unit vector along z-axis, as illustrated in Figure 1. In a more physical perspective, Equation (1) means the momentum mismatch between the forward and backward propagating modes should be compensated by the reciprocal vector provided by the periodical index modulation. For the first-order diffraction which is commonly seen in MFBGs:

(2) $$\left(n_{\text{eff},f}+n_{\text{eff},b}\right)\Lambda =\lambda_{B}.$$

Furthermore, if the two modes are identical, the commonly used Bragg resonance condition can be obtained, namely, λ_B_ = 2n_eff_Λ.

Before designing the grating, n_eff_ should be calculated first. For a uMFBG, the index modulation is relatively weak (usually on the order of 10^−4^) and the cross section of the MF is a symmetrical circle. n_eff_ can be easily obtained solving the dispersion equations numerically.

**Figure 4.**
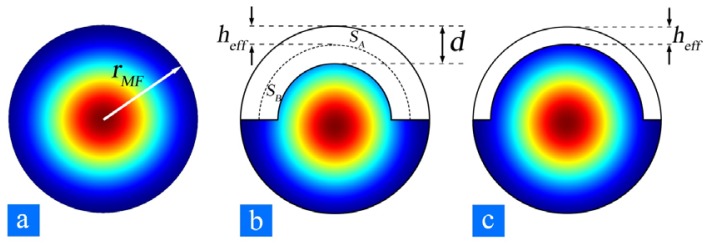
The cross-section and fundamental modal field distribution of (**a**) an un-milled MF, (**b**) a milled MF and (**c**) an equivalent unperturbed geometry, respectively. d is the depth of the corrugation and h_eff_ is the boundary shift. S_A_(S_B_) is the area bounded by the upper(lower) part of the corrugation. Modal fields are calculated for r_MF_ = 2 μm, d = 200 nm, and h_eff_ = 97.4 nm.

However, for a sMFBG, the effective index difference between the MF milled and un-milled cross section can be as large as ∼10^−3^ [31], or even ∼10^−1^ [34], orders of magnitude larger than that in conventional FBGs. One way to calculate an averaged effective index of the grating region is to choose an unperturbed waveguide boundary [48], shown as the curved dashed line in Figure 4(b). d is the depth of the corrugation and h_eff_ is the boundary shift from the top of the corrugation to the new boundary of the corresponding unperturbed waveguide. The boundary shift h_eff_ as illustrated in Figure 4(b,c) is determined such that the volume bounded by the upper part of the corrugation (S_A_τ) is equal to that of the volume bounded below [S_B_(1−τ)], *i.e.*,

(3) $$S_{A}\tau =S_{B}(1-\tau ).$$

where τ is the duty cycle. By assuming that τ = 0.5 and d ≪ r_MF_, we get h_eff_ ≈ d/2. The averaged effective index could thus be obtained by mode analysis after the unperturbed waveguide boundary is established.

The uMFBGs reflection spectrum can be estimated using [49]

(4) $$R=\frac{{\text{sinh}}^{2}\left(\sqrt{\kappa^{2}-\gamma^{2}}L\right)}{{\text{cosh}}^{2}\left(\sqrt{\kappa^{2}-\gamma^{2}}L\right)-\frac{\gamma^{2}}{\kappa^{2}}}.$$

where κ, γ, L is the coupling coefficient, self-coupling coefficient, and length of the grating, respectively. Due to the weak index modulation seen in the uMFBGs, MFs with grating region up to several hundreds of micrometers or millimeters in length is needed in order to get detectable reflection [22,23,26].

**Figure 5.**
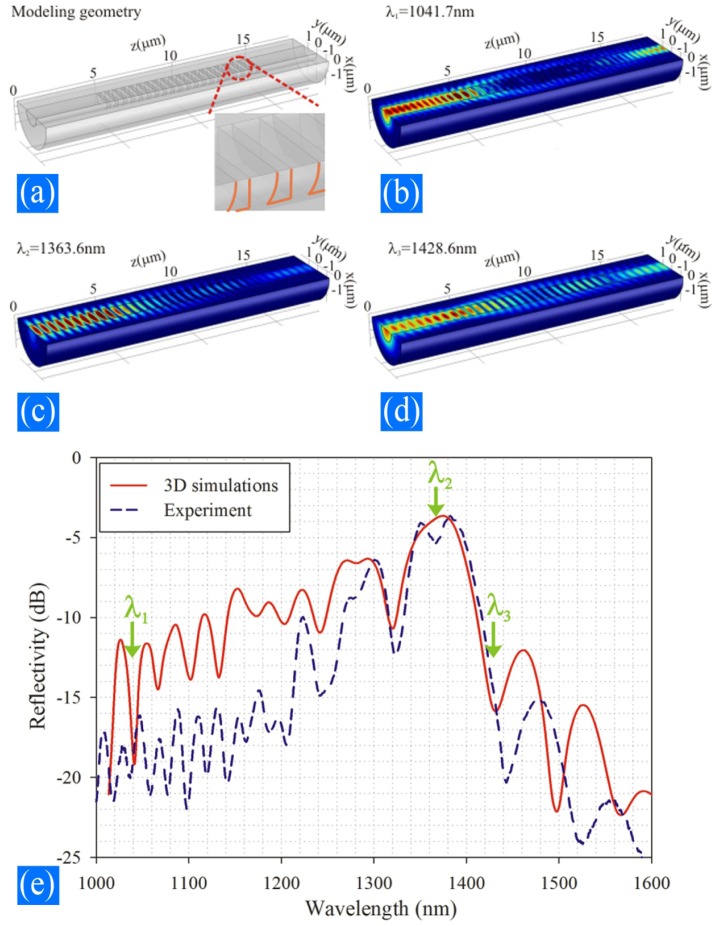
(**a**) Schematic of the sMFBG modeling; the insert shows the magnified figure of the biconcave air notch. (**b**), (**c**), and (**d**) Electric fields at wavelength λ_1_ = 1,041.7 nm, λ_2_ = 1,363.6 nm, and λ_3_ = 1,428.6 nm, respectively. (**e**) MFBG reflection spectra. The red solid line is the 3D simulation line while the blue dashed line is the experiment result. λ_1_, λ_2_, and λ_3_ represent the wavelengths whose electric fields are shown in (**b**), (**c**), and (**d**).

However, due to the large index difference in the corrugation region in sMFBGs, strong scattering may occur. A more effective way to verify the experimental spectrum obtained from the sMFBG is a 3D finite element simulation, shown in Figure 5. This method takes the details of the structural deformation into consideration and thus better reflects the real situation experienced by the light field.

### 3.2. Sensing Properties of MFBGs

Most MFBG sensors rely on the monitoring of the shift in wavelength of the reflected Bragg signal with the changes in the measurand (e.g., refractive index, temperature, and strain/force).

As the effective index and period of grating is a function of r_MF_, n_a_, T, and ε, the Bragg condition can be rewritten as

(5) $$\lambda_{B}=2n_{\text{eff}}(r_{MF},n_{f},n_{a},T,\varepsilon )\Lambda (T,\varepsilon ).$$

where the RI of the fused silica and the ambient medium surrounding the MFBG are denoted by n_f_ and n_a_, T is the operating temperature and ε is the strain applied to the MFBG.

#### 3.2.1. Refractive Index Sensing

The notable distinction between MFBGs and conventional FBGs lies in that the MFBG large evanescent field which enables its capabilities for external medium sensing. When a MFBG is operated as a RI sensor, the wavelength shift depends on the change of n_a_. Sensitivity with respect to the ambient medium RI (S_a_) is defined as

(6) $$S_{a}=\frac{d\lambda_{B}}{dn_{a}}=\frac{\partial \lambda_{B}}{\partial n_{\text{eff}}(n_{a},r_{MF})}\frac{\partial n_{\text{eff}}(r_{MF},n_{a})}{\partial n_{a}}=2\Lambda \frac{\partial n_{\text{eff}}}{\partial n_{a}}.$$

**Figure 6.**
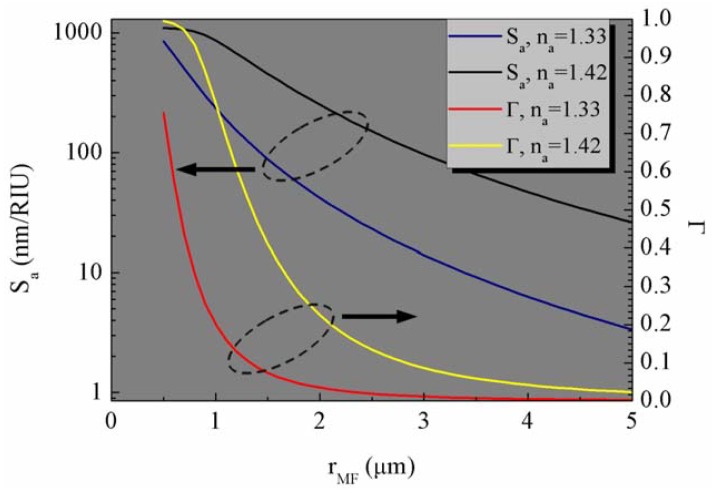
Sensitivity S_a_ (blue and black line) and power fraction propagating in the surrounding medium Γ (red and yellow line) for different MF radii r_MF_ in the range 0.5–5 μm. Λ is set at 550 nm and n_a_ is chosen to be at 1.33 and 1.42. Only the fundamental mode is considered.

Figure 6 shows S_a_ and the power fraction propagating in the ambient medium (Γ) as a function of r_MF_. n_a_ is chosen to be 1.33 and 1.42, because most of the RI sensors work around these values. Both Γ and S_a_ increase for decreasing MF diameters, which indicates larger fractions of power propagating in the evanescent field, thus in the surrounding environment. When the MF diameter reaches 1 μm, 75% of the energy propagates outside the MF for n_a_ = 1.33, whereas Γ ∼99.5% for n_a_ = 1.42. In addition, a larger S_a_ is associated to higher external RIs. Theoretically, the largest S_a_ which can be obtained by MFBG is 2Λ, typically around 1100 nm/RIU according to Equation (6). This value is comparable to optical microfiber coil resonator sensor [6,7] and higher than microcapillary resonator [50] or photonic crystal microresonator [51].

#### 3.2.2. Temperature Sensing

Temperature affects the Bragg wavelength shift through the thermo-optical and thermal expansion effects in three ways: index variation, MF radius variation and the grating period change, each of which is represented in Equation (7). The temperature sensitivity (S_T_) can be defined as:

(7) $$S_{T}=\frac{d\lambda_{B}}{dT}=2\Lambda \left(\sigma_{T}\frac{\partial n_{\text{eff}}}{\partial n_{f}}+r\alpha_{T}\frac{\partial n_{\text{eff}}}{\partial r}+n_{\text{eff}}\alpha_{T}\right)$$

Here, σ_T_ (1.2 × 10^−5^/°C) is the thermo-optical coefficient and α_T_ (5.5 × 10^−7^/°C) is the thermal expansion coefficient of fused silica. As thermal expansion contributes less than 2 pm/°C to the total sensitivity, it is generally neglected. S_T_ resulting from the thermo-optical effect is ∼10–20 pm/°C and dominates in temperature sensing, which is in agreement with previous results using fiber tip Febry-Perot interferometer [9].

#### 3.2.3. Strain/Force Sensing

From the continuum mechanics, when longitudinal strain is applied to a MFBG, wavelength shift can be estimated as follows:

(8) $$\Delta \lambda_{B}=2\Lambda n_{\text{eff}}\;\left\{1-\left(\frac{n_{\text{eff}}^{2}}{2}\right)\;\left[p_{12}-\nu (p_{11}+p_{12})\right]\right\}\varepsilon =2\Lambda n_{\text{eff}}\;\left(1-p_{\text{eff}}\right)\varepsilon ,$$

where ε is the applied strain, ν is Poisson's ratio and p_ij_ coefficients are the Pockel's strain-optical tensor of the fiber material. Here, the structural deformation of the MF cross section due to the applied stain is neglected. Consequently, the strain sensitivity (S_S_) is reduced to:

(9) $$S_{S}=\frac{\Delta \lambda_{B}}{\varepsilon }=\lambda_{B}\left(1-p_{\text{eff}}\right).$$

The effective photo-elastic coefficient p_eff_ for a MFBG strain sensor is ∼0.21, giving S_S_ ∼1.2 pm/με which is compared to that of a conventional fiber at a Bragg wavelength of 1550 nm. This is in agreement with experimental results [24,26]. Another way to characterize the capability of MFBG sensors is to use the force sensitivity (S_F_):

(10) $$S_{F}=\frac{S_{S}}{\pi r_{MF}^{2}E},$$

with E representing Young's modulus. Equation (10) shows that S_F_ scales inversely with the square of the microfiber diameter.

## 4. Sensing Applications

### 4.1. Refractive Index Sensing

Much of the MFBG applications relate to RI sensing. For a typical MFBG sensor immersed in ambient liquid with RI in the range 1.32–1.46, S_a_ varies from 10^1^ nm/RIU (refractive index unit) to 10^3^ nm/RIU, according to the MF radius and the ambient liquid sensed. Usually, a smaller radius and a larger ambient medium RI result in a higher sensitivity regardless of the fabrication method. For example, Liang *et al.* got a sensitivity of 16 nm/RIU at a RI around 1.35 with a MF 6 μm in diameter [22] while 660 nm/RIU was reached by Liu *et al.* at a RI of 1.39 by using a 1.8 μm-diameter MF [36]. Both of them agree well with what is predicted from Equation (6). Significant results previously reported in the literature are listed in Table 1.

**Figure 7.**
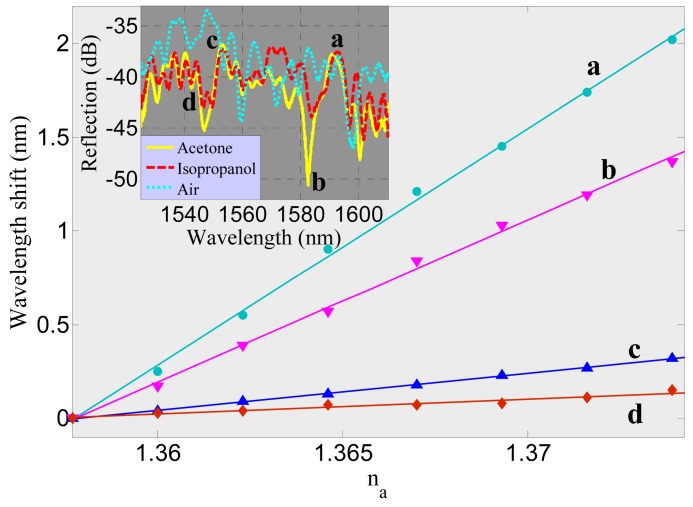
Dependence of wavelength shift on ambient RI for different modes in a metal-dielectric-hybrid grating. a, b, c, d denote different peaks and valleys labeled in the inset figure. Marks represent experimental results while solid lines are linear fittings. Inset: measured reflection spectra of the metal-dielectric-hybrid grating when immersed in air, acetone, and isopropanol.

In addition to all these nonmetallic MFBGs, metallic gratings have also been proposed for RI sensing. The existence of metal causes light to be coupled to modes of different properties [32,42]. A metal-dielectric-hybrid grating (Figure 2(b)) showed RI sensitive (a in Figure 7) and insensitive (d in Figure 7) behavior for different resonant modes (see inset of Figure 7) [32]. S_a_ of the sensitive channel (125 nm/RIU) is one order of magnitude larger than that of a nonmetallic MFBG with the same radius whereas S_a_ of the insensitive channel (8 nm/RIU) is one order of magnitude smaller. This can be attributed to the fact that the introduction of metal film causes the MF to support both surface guided modes (which have a larger modal overlap with the ambient medium, Figure 7(a,b)) and bound modes (where most of the energy is located in the dielectric core, Figure 7(c,d)).

### 4.2. Temperature Sensing

**Figure 8.**
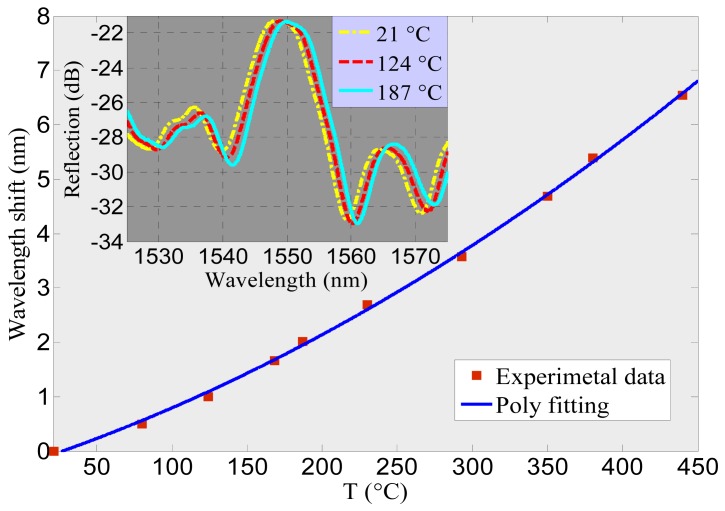
Dependence of the measured wavelength shift on temperature T. Inset: reflection spectra of the MFBG in air at three different temperatures.

Although thermal post-processing and hydrogen loading have been shown to induce grating capable of standing temperatures as high as 1,300 °C in conventional fibers [52]; for uFMBGs, only small temperature ranges have been detected because the photosensitized index modulation is unstable at high temperatures. In MF thermometers, up to now, only sMFBGs have been reported operating above 200 °C [31,35]. The sensitivity of these components is around 20 pm/°C, similar to the value predictable using Equation (7). Figure 8 is the experimental characterization of the sFMBG demonstrated using the sample shown in Figure 2(a). As the temperature increases, the Bragg wavelength red shifts. The extremely short length of the MFBG (∼36.6 μm) and wide operating range (∼20–450 °C) presents it a promising candidate for detecting temperature change in ultra-small spaces.

### 4.3. Strain/Force Sensing

Although S_S_ remains almost the same for different MF diameters [26], S_F_ varies with the MF radius according to Equation (10). A MFBG with diameter of 3.5 μm reaches a force sensitivity of ∼1900 nm/N, which is more than three orders of magnitude compared to that of a conventional fiber [26]. If the Bragg wavelength can be detected to an accuracy of 0.05 nm, it would be possible to measure forces in the order of 10^−5^ N. For the sample reported in Figure 2(d), where the silica constitute only a small fraction of the MFBG cross section, a further three orders of magnitude improvement in sensitivity is predicted, with S_F_ reaching values in excess of 10^6^ nm/N, corresponding to forces of the order of nN. The MFBG strain/force sensors could offer attractive properties monitoring strain/force changes in power plant pipelines, airplane wings, and other civil engineering structures.

## 5. Conclusions and Outlook

MFBGs can potentially outperform conventional FBGs because of their large evanescent field and compactness. This review presented the fabrication, operating principles and applications of MFBGs. Due to their ultra-small size (especially the sMFBGs), MFBGs could find promising sensing applications in detecting parameter variations in ultra-small spaces.

Future work may focus on (1) expanding the MFBG to simultaneous multi-parameter measurement, such as using the metal-dielectric-hybrid grating; (2) utilizing MFBG to operate in extreme temperatures as high as 1,500 °C; (3) studying sMFBG for strain/force sensing applications; (4) taking advantage of other materials to increase S_T_, which is now mainly limited by the thermo-optical coefficient of silica.

## Figures and Tables

**Table 1.** A summary of MFBG sensors reported in the literature.

**Measurand, Fabrication method**	**Sensitivity** **(nm/RIU or pm/°C or pm/με)**	**Measured range** **(RIU or °C)**	**Length of grating** **(μm)**	**Radius of MFs** **(μm)**	**Ref.**
RI, Etch-eroded MF and UV irradiated FBG	16 nm/RIU @ a RI around 1.35	1–1.378	2,500	3.0	[22]
RI, Etch-eroded MF and commercial FBG	--	1.35–1.42	700	5.3	[23]
RI, MF by flame brushing method and FIB-milled FBG	660 nm/RIU @ a RI of 1.39	1.33–1.39	518	0.9	[36]
RI, Suspended core fiber drawn from preform and UV irradiated FBG	∼167 nm/RIU @ a RI of 1.40	∼1.40–1.41	--	1.7	[43]
RI, MF wrapped on a microstructured rod	1,200 nm/RIU @ a RI of 1.33	--	--	0.3	[41]
RI, Etch-eroded MF and commercial FBG	1st mode: 19.4 2nd mode: 52.1 3rd mode: 92.0 nm/RIU @ a RI around 1.38	∼1.32–1.41	50	3.5	[24]
RI, MF by commercial puller and FIB-milled FBG	125 nm/RIU @ a RI around 1.36	1.358–1.374	10	3	[32]
RI, FBG by metal lift-off technology	511 nm/RIU @ a RI around 1.41	1.00–1.42	5,000	5	[42]
RI, MF by flame brushing method and FBG by femtosecond laser pulse irradiation	231.4 nm/RIU @ a RI of 1.44	1.32–1.46	4,000	1	[40]
Temperature, MF by commercial puller and FIB-milled FBG	20	21–440	36.6	3.3	[31]
Temperature, MF by commercial puller and FIB-milled FBG	22	23–228	12	∼2.5	[35]
Temperature, Etch-eroded MF and commercial FBG	1st mode: 13.0 2nd mode: 15.9 3rd mode: 32.0	30–60	50	3.5	[24]
Strain/Force, MF by CO_2_ laser heating technique and UV irradiated FBG	∼1.2	0–0.15N	5,000	1.75	[26]
Strain, Etch-eroded MF and commercial FBG	0.9	--	50	3.5	[24]
